# Advances in the Identification of Novel Urinary Biomarkers for Diabetic Kidney Disease

**DOI:** 10.1155/jdr/7990072

**Published:** 2026-07-23

**Authors:** Yujie Jin, Yan Ma, Yan Yao, Mengru Wang, Chunchen Ni, Shujuan Shang, Yongxin Cui, Xinyu Wang, Ye Ling, Yumeng Sun, Qirui Pei, Shiqiang Liu, Lizhuo Wang, Jialin Gao

**Affiliations:** ^1^ Department of Endocrinology and Genetic Metabolism, The First Affiliated Hospital of Wannan Medical University (Yijishan Hospital of Wannan Medical University), Wuhu, Anhui, China; ^2^ Institute of Endocrine and Metabolic Diseases, The First Affiliated Hospital of Wannan Medical University (Yijishan Hospital of Wannan Medical University), Wuhu, Anhui, China; ^3^ Anhui Province Key Laboratory of Basic Research and Transformation of Age-Related Diseases, Wannan Medical University, Wuhu, Anhui, China; ^4^ Department of Biochemistry and Molecular Biology, Wannan Medical University, Wuhu, Anhui, China

**Keywords:** diabetic kidney disease, noninvasive detection, research progress, urinary metabolic markers

## Abstract

Diabetic kidney disease (DKD) is a major microvascular complication of diabetes and remains one of the leading causes of end‐stage renal disease, significantly affecting patients′ survival rates and quality of life. Currently, commonly used clinical assessment indicators include proteinuria and estimated glomerular filtration rate (eGFR); however, these indicators have limited sensitivity, making it difficult to detect early kidney damage in a timely manner and to accurately monitor disease progression. This review provides a comprehensive overview of recent progress in identifying various urinary biomarkers that reflect renal tubular injury, oxidative stress, inflammatory responses, fibrotic remodeling, metabolic dysregulation, and exosomal components. Furthermore, we discuss the potential clinical applications of these biomarkers in DKD early diagnosis, disease stratification, and prognostic evaluation. Looking ahead to the future and the ongoing development of multiomics integration and artificial intelligence–assisted modeling, urinary biomarkers are expected to drive DKD diagnosis and management toward a future characterized by early detection, precision, dynamic monitoring, and noninvasive assessment.

## 1. Introduction

Diabetic kidney disease (DKD) is a major microvascular complication of diabetes and remains one of the leading causes of end‐stage renal disease, significantly affecting patients′ survival rates and quality of life. Currently, commonly used clinical assessment indicators include proteinuria and estimated glomerular filtration rate (eGFR); however, these indicators have limited sensitivity, making it difficult to detect early kidney damage in a timely manner and to accurately monitor disease progression [1]. Although current treatment strategies, including glycemic control and antihypertensive therapy, can partially delay the progression of DKD, significant limitations remain in routine clinical practice. At present, the early identification of DKD still relies primarily on traditional markers such as the urine albumin‐to‐creatinine ratio (UACR) and eGFR. However, these parameters typically only become abnormal once structural kidney damage has become quite severe. Furthermore, both UACR and eGFR have limited sensitivity and specificity for detecting early and dynamic changes in renal function, which restricts their utility in timely risk stratification and intervention. These limitations highlight the need to explore more sensitive and mechanistically informative biomarkers to enable the early detection and more precise monitoring of DKD.

Moreover, the recent advancement of omics technologies and high‐throughput analytical platforms has shifted research focus toward urinary biomarkers, which hold considerable promise for the early screening of DKD. Urine offers a noninvasive, easily accessible, and biologically informative specimen to measure DKD and offers unique advantages for monitoring metabolic changes, renal injury, and associated pathological processes [2]. The emergence of metabolomics and proteomics has further driven the identification of novel urinary biomarkers. For example, glycogen synthase kinase 3*β* (GSK‐3*β*), high mobility group box 1 (HMGB1) protein, insulin‐like growth factor binding proteins (IGFBPs), and urinary exosomes are among the specific metabolites thought to be closely associated with the onset, progression, and underlying mechanisms of DKD. These biomarkers not only capture early and subtle renal injury but also offer significant value in disease prediction, clinical monitoring, personalized treatment, and prognostic evaluation [3].

This review is aimed at providing recent advances in urinary biomarkers for DKD, highlighting their roles in early diagnosis, mechanistic insight, and clinical intervention. Additionally, we discuss the current clinical application challenges and future directions in the potential translation of these biomarkers.

## 2. Renal Injury and Functional Biomarkers

During the early stages of DKD, one of the earliest pathological changes is renal tubular injury, and at that point, various urinary biomarkers often emerge as sensitive and noninvasive indicators of this process. Molecules such as kidney injury molecule‐1 (KIM‐1), N‐acetyl‐*β*‐D‐glucosaminidase (NAG), neutrophil gelatinase‐associated lipocalin (NGAL), cystatin C, *β*2‐microglobulin, *α*1‐microglobulin, and alkaline phosphatase (ALP) demonstrate high sensitivity for detecting tubular damage. In addition to markers of tubular structural damage, a range of glomerular biomarkers, including transferrin, uPAR/suPAR, and B7‐1 (CD80), have also been found to be associated with early alterations in the glomerular filtration barrier in DKD. These molecules are closely linked to podocyte dysfunction and disruption of the slit‐pore membrane integrity, which together lead to increased glomerular permeability as the disease progresses. Biomarkers associated with tubular metabolism and repair—such as GSK‐3*β*, soluble cMet (s‐cMet), calmodulin 1 (CALM1), epidermal growth factor (EGF), citrate, and inositol—often occur in parallel and reflect the dynamic regulatory changes in tubular epithelial cells that occur in response to metabolic stress, apoptosis, and tissue repair in the diabetic milieu. The analysis of injury‐related and function‐related biomarkers offers a promising strategy for improving early diagnosis, assessing disease progression, and monitoring therapeutic efficacy in DKD.

### 2.1. Glomerular and Tubular Injury Biomarkers

KIM‐1 is a Type I transmembrane glycoprotein that is strongly induced following damage to proximal tubular epithelial cells. In the context of DKD, elevated levels of KIM‐1 in urine reflect persistent stress on the tubular epithelium and indicate that repair programs following diabetic metabolic injury have been activated. Functionally, KIM‐1 is involved in the clearance of apoptotic cells and in epithelial remodeling processes, both of which are crucial for tissue repair following acute injury.

However, under conditions of persistent hyperglycemia, prolonged activation of the KIM‐1‐mediated repair pathway may result in maladaptive responses, leading to tubular dedifferentiation, atrophy, and interstitial fibrotic remodeling, thereby accelerating the progression of chronic kidney disease. Clinically, KIM‐1 has been demonstrated to be a sensitive marker of early tubular damage in DKD, particularly in patients who have not yet developed overt proteinuria or a significant decline in eGFR. Furthermore, circulating and urinary KIM‐1 levels correlate positively with the deterioration of renal function and the extent of tubulointerstitial damage, supporting its significant value in early diagnosis and longitudinal monitoring of the disease [4–7]. NAG, a lysosomal enzyme derived from renal tubular epithelial cells, provides evidence of tubular injury when released into the urine. Moreover, urinary NAG levels appear significantly elevated in diabetic patients, especially during the early stages of nephropathy. This supports its potential utility as a noninvasive early diagnostic marker for DKD [8, 9]. Under conditions of oxidative stress and inflammatory activation, NGAL is induced in renal tubular epithelial cells, reflecting the stress response program in the early stages of DKD. Functionally, NGAL is involved in iron transport and tubular stress adaptation and is closely associated with the progression of tubulointerstitial damage.

In DKD, urinary NGAL levels rise in parallel with the severity of proteinuria and a decline in eGFR, indicating its association with overall renal functional impairment. It is worth noting that NGAL exhibits dynamic changes at different stages of the disease: Mild elevations may occur during the early phase of glomerular hyperfiltration, reflecting subclinical tubular stress; whereas in later stages, when tubulointerstitial damage is established and renal function gradually deteriorates, driven by persistent inflammation and oxidative stress, NGAL levels remain persistently elevated.

Overall, NGAL reflects both early tubular stress and the progression of persistent damage, supporting its role as a dynamic biomarker for DKD staging and prognostic stratification [8, 10–13].

Furthermore, cystatin C, a low‐molecular‐weight cysteine protease inhibitor, is freely filtered by the glomerulus and reabsorbed in the renal tubules. Elevated urinary levels of cystatin C are indicative of impaired tubular reabsorption and have been closely linked to early tubular dysfunction and declining renal function in DKD; therefore, cystatin C can be a useful early marker of renal injury [8, 14]. In addition, *β*2‐microglobulin is a small protein normally filtered by the glomerulus and nearly entirely reabsorbed in the proximal tubules. In DKD, increased urinary *β*2‐microglobulin reflects proximal tubular dysfunction and is considered a sensitive biomarker for early renal damage [15]. Similarly, *α*1‐microglobulin, another low‐molecular‐weight protein, is reabsorbed by healthy renal tubules; therefore, elevated urinary *α*1‐microglobulin levels are indicative of tubular dysfunction and are associated with early‐stage renal impairment in patients with diabetes. These findings underscore the potential for *α*1‐microglobulin to be used as a diagnostic tool in DKD [16]. ALP, a hydrolytic enzyme primarily localized to the brush border of proximal tubular epithelial cells, is also released into the urine in response to tubular epithelial damage. Elevated urinary ALP levels are correlated with urinary albumin excretion and can often appear before measurable declines in eGFR. These details suggest the potential role that ALP can play in early detection of DKD [17–19].

In addition to markers of tubular damage, certain glomerular biomarkers have also been found to be associated with early glomerular changes in DKD. Among these, urinary transferrin is closely associated with increased glomerular permeability and endothelial dysfunction, and its elevation may occur even before the onset of overt microalbuminuria. These findings suggest that transferrin may be capable of detecting early microvascular damage to the glomerular filtration barrier, thereby offering additional value for the early detection of DKD [20]. The uPAR/suPAR axis is associated with podocyte damage, glomerulosclerosis, and the progressive deterioration of renal function in DKD. Experimental studies have shown that suPAR can induce podocyte migration and apoptosis via integrin‐mediated signaling pathways, disrupting cytoskeletal stability and compromising the integrity of the glomerular filtration barrier, thereby accelerating the progression of DKD [21]. B7‐1 (CD80) is an immunostimulatory molecule associated with podocyte dysfunction and proteinuric nephropathy. In podocytes, stress‐induced expression of B7‐1 is thought to disrupt the structure of the gap junctions and impair podocyte adhesion signaling pathways, thereby compromising the glomerular filtration barrier and leading to proteinuria [22].

Biomarkers of glomerular and tubular damage collectively reflect the complex pathological features of DKD, including tubular epithelial stress, impaired reabsorption, podocyte dysfunction, and disruption of the glomerular filtration barrier. It is worth noting that changes in some of these biomarkers may occur before the onset of overt albuminuria and a measurable decline in eGFR, suggesting that they have the potential to reflect early microstructural changes in the kidney and may aid in a more refined assessment of the risk of DKD.

### 2.2. Tubular Metabolism, Inflammation, and Repair

Moreover, GSK‐3*β* is a multifunctional serine/threonine kinase involved in regulating glucose metabolism, cellular repair, and renal tubular epithelial cell apoptosis. In patients with DKD, urinary GSK‐3*β* activity is significantly elevated and correlates with the severity of proteinuria and declining renal function. These findings suggest GSK‐3*β*′s potential utility as a noninvasive biomarker of metabolic dysregulation and impaired tubular repair. Furthermore, inhibition of GSK‐3*β* can attenuate renal inflammation and fibrosis, highlighting its value as a potential therapeutic target in DKD [23, 24]. Hepatocyte growth factor (HGF) and its receptor cMet play key roles in tissue repair and antifibrotic processes. For example, s‐cMet, a cleavage product released under tubular epithelial injury, is elevated in DKD patients′ urine. Urinary s‐cMet levels are also positively correlated with proteinuria and renal function decline, indicating its promise as a biomarker for early tubular injury and repair. Moreover, the HGF/cMet signaling axis may reflect endogenous antifibrotic mechanisms activated during disease progression [25]. The calcium‐binding messenger protein, CALM1, is highly expressed in renal tubular cells and plays an essential role in calcium signaling pathways. Recent studies suggest elevated CALM1 levels in the urinary exosomes of patients with DKD. These levels also correlate with renal dysfunction. These data suggest that CALM1 may reflect cellular stress and compensatory repair responses in tubular epithelial cells that are under metabolic and inflammatory stress [26–30]. Moreover, EGF, which is primarily synthesized by renal tubular epithelial cells, is crucial for tubular regeneration, antiapoptotic signaling, and repair processes. Urinary EGF levels in DKD patients are markedly reduced and negatively associated with proteinuria, renal function decline, and tubulointerstitial fibrosis. Notably, the urinary EGF‐to‐monocyte chemotactic protein‐1 (MCP‐1) ratio could be a predictor of rapid DKD progression, offering both diagnostic and prognostic value [28, 31–34].

Further, citrate acts as a key intermediate in the tricarboxylic acid (TCA) cycle in tubular cells and reflects mitochondrial metabolic status and acid–base balance. Studies suggest that urinary citrate levels are reduced in patients with DKD and are closely associated with tubular metabolic stress, mitochondrial dysfunction, and renal deterioration [35]. Myo‐inositol, a polyol derived from glucose metabolism, is primarily synthesized in renal tubular epithelial cells and plays a vital role in osmotic regulation, signal transduction, and cellular homeostasis. Further, elevated myo‐inositol urinary levels are observed in DKD, which likely reflects impaired tubular metabolic regulation in response to hyperglycemia. Importantly, these changes may occur before eGFR decline, highlighting myo‐inositol′s potential as an early biomarker of tubular metabolic stress [36]. Overall, biomarkers involved in tubular metabolism and repair reflect key aspects of the dynamic metabolic reprogramming and regenerative responses of tubular epithelial cells during the progression of DKD. These molecules capture a range of damage signatures, including mitochondrial dysfunction, cellular stress responses, and impaired repair capacity, and may provide early signals of metabolic disturbances prior to the onset of overt structural kidney damage. Table 1 summarizes representative urinary biomarkers associated with glomerular and tubular damage, impaired tubular repair, and metabolic stress in DKD.

**Table 1:** Renal injury and tubular metabolism–associated biomarkers in DKD.

Biomarker name	Function	Clinical significance (EN)	Reference
Glomerular and tubular injury biomarkers			
KIM‐1	Type I transmembrane glycoprotein involved in tubular epithelial repair and regeneration	Reflects early tubular injury, maladaptive repair, and progression of DKD	[4]
NAG	Lysosomal enzyme released from proximal tubular epithelial cells	Indicates proximal tubular injury and early renal damage in DKD	[8]
NGAL	Acute tubular injury and oxidative stress–associated protein	Reflects tubular injury, inflammation, and progression of renal dysfunction in DKD	[10]
Cystatin C	Low‐molecular‐weight protease inhibitor filtered by the glomeruli and reabsorbed by the tubules	Indicates glomerular filtration dysfunction and tubular impairment	[14]
*β*2‐Microglobulin	Low‐molecular‐weight protein reabsorbed by proximal tubules	Reflects tubular reabsorption dysfunction and early renal injury	[15]
*α*1‐Microglobulin	Low‐molecular‐weight glycoprotein associated with tubular reabsorption	Indicates proximal tubular dysfunction and early DKD progression	[16]
Tubular metabolism and repair biomarkers			
ALP	Brush‐border enzyme of proximal tubular epithelial cells	Reflects proximal tubular epithelial damage in DKD	[17]
Transferrin	Glomerular permeability–associated protein	Indicates early glomerular filtration barrier dysfunction and endothelial injury	[20]
uPAR	Regulator of cell adhesion and podocyte signaling pathways	Associated with podocyte injury, glomerulosclerosis, and DKD progression	[21]
B7‐1	Immune costimulatory molecule involved in podocyte activation	Reflects podocyte dysfunction and glomerular filtration barrier injury	[22]
GSK‐3*β*	Serine/threonine kinase regulating metabolism, apoptosis, and fibrosis	Reflects metabolic dysregulation, tubular injury, and fibrosis progression	[23]
HGF	Growth factor involved in tissue repair and antifibrotic signaling	Indicates tubular repair activity and endogenous antifibrotic responses	[25]
s‐cMet	Soluble cleavage product of the HGF receptor cMet involved in tubular repair and antifibrotic signaling	Elevated urinary s‐cMet reflects tubular epithelial injury, impaired repair responses, and progressive renal dysfunction in DKD	[25]
Calcineurin	Calcium/calmodulin‐dependent phosphatase involved in cellular signaling	Reflects calcium signaling abnormalities and renal cellular stress in DKD	[26]
EGF	Tubular epithelial–derived growth factor involved in regeneration and repair	Reduced urinary EGF reflects impaired tubular repair and progressive fibrosis	[28]
Citrate	Intermediate metabolite of the tricarboxylic acid (TCA) cycle	Reflects mitochondrial dysfunction, tubular metabolic stress, and renal deterioration	[35]
Myo‐inositol	Polyol metabolite involved in osmotic regulation, signal transduction, and tubular cellular homeostasis	Elevated urinary myo‐inositol reflects tubular metabolic dysregulation, hyperglycemia‐associated stress, and early mitochondrial metabolic impairment in DKD	[36]

## 3. Markers of Oxidative Stress and Inflammation

Oxidative stress and inflammatory activation are closely associated with the onset and progression of DKD, jointly leading to tubular damage, endothelial dysfunction, immune activation, and a sustained decline in renal function. In a hyperglycemic environment, enhanced glucose metabolism and mitochondrial dysfunction promote the production of excessive reactive oxygen species (ROS), thereby causing oxidative damage to DNA and lipids and triggering downstream inflammatory signaling pathways.

Urinary biomarkers associated with oxidative stress, such as 8‐hydroxy‐2 ^′^‐deoxyguanosine (8‐OHdG), malondialdehyde (MDA), superoxide dismutase (SOD), and mitochondrial glutathione S‐transferase (Mu‐GST), primarily reflect the extent of oxidative damage and antioxidant capacity, whereas inflammatory mediators such as interleukin‐6 (IL‐6), MCP‐1, soluble CD163 (sCD163), and soluble CD40 ligand (sCD40L) reflect a state of persistent immune activation within the renal microenvironment.

**Figure 1:**
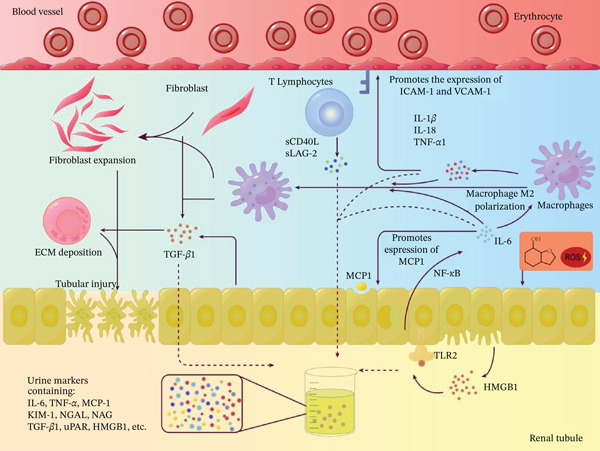
Mechanistic interactions among oxidative stress, inflammatory activation, tubular injury, and fibrosis during DKD progression. Under hyperglycemic conditions, excessive glucose metabolism and mitochondrial dysfunction induce reactive oxygen species (ROS) overproduction, thereby promoting oxidative stress and tubular epithelial injury. Persistent oxidative stress further activates HMGB1/TLR/NF‐*κ*B signaling pathways and enhances the release of inflammatory cytokines and chemokines, including IL‐6, IL‐1*β*, IL‐18, TNF‐*α*, and MCP‐1, which subsequently promote macrophage recruitment and inflammatory amplification. Sustained inflammatory activation further contributes to TGF‐*β*1‐mediated fibroblast activation, ECM deposition, and progressive tubulointerstitial fibrosis. These pathological alterations are accompanied by increased urinary excretion of multiple biomarkers, including 8‐OHdG, MDA, HMGB1, MCP‐1, NGAL, KIM‐1, TGF‐*β*1, and uPAR, which may reflect different pathological stages of DKD progression, ranging from early oxidative injury and glomerular hyperfiltration to advanced fibrosis and irreversible renal damage.

These molecular markers exhibit dynamic and stage‐dependent characteristics during the progression of DKD. In the early stages of the disease, elevated levels of oxidative stress markers such as 8‐OHdG and MDA are observed, suggesting the presence of subclinical redox imbalance prior to marked functional decline. As the disease progresses to tubulointerstitial damage, inflammatory mediators including MCP‐1, IL‐6, interleukin‐18 (IL‐18), HMGB1, sCD163, and sCD40L gradually increase, reflecting ongoing interactions between the immune system and the renal tubules. In advanced DKD, fibrosis‐related signals such as transforming growth factor‐*β*1 (TGF‐*β*1), miR‐21, and circulating mitochondrial DNA (mtDNA) predominate, corresponding to irreversible structural remodeling and renal failure. Figure 1 illustrates the coordinated interactions between oxidative stress, inflammation, tubular damage, and fibrotic remodeling in DKD.

### 3.1. Oxidative Stress and DNA Damage

Urinary 8‐OHdG, a product of oxidative DNA damage, is a well‐recognized marker of oxidative stress. As such, elevated urinary 8‐OHdG levels are observed in patients with DKD, particularly those with overt proteinuria. This suggests that renal oxidative stress may contribute to kidney function deterioration [37]. Further, thiobarbituric acid reactive substances (TBARS) reflect lipid peroxidation mediated by free radicals. For example, reactive carbonyl derivatives (RCDs) are products of oxidized protein carbonyl groups, and sulfur‐containing antioxidants under oxidative stress are reduced into total sulfhydryl groups (TSHGs). These measures collectively provide a comprehensive evaluation of the severity of oxidative damage in patients with DKD [38]. Furthermore, glutathione S‐transferase (GST), a key detoxifying enzyme involved in redox homeostasis, has been increasingly recognized in DKD‐related injury. Recent studies suggest that Wilms tumor protein 1 (WT‐1) and Mu‐GST are promising urinary biomarkers showing significant correlations with the albumin‐to‐creatinine ratio (ACR). Codetection of WT‐1 and Mu‐GST enhances the accuracy of tubular injury assessment as well, as they are indicative of distal tubular damage [39]. SOD, an essential antioxidant enzyme, plays a central role in scavenging superoxide anions and maintaining oxidative balance. Elevated serum and urinary levels of extracellular SOD3 have been reported in DKD patients, as well as higher urinary albumin and serum creatinine levels. These findings suggest that urinary SOD may offer valuable insights into renal oxidative stress and disease progression [40].

Uric acid, the final product of purine metabolism, is often associated with tubular dysfunction when altered in urinary excretion. Beyond its metabolic role, uric acid has also been identified as a marker of DKD pathogenesis of DKD through mechanisms involving oxidative stress and inflammation. Notably, decreased urinary uric acid excretion (UUAE) is linked to a heightened risk of chronic kidney disease in patients with Type 2 diabetes [41]. Further, MDA, a well‐established end product of lipid peroxidation, is widely used as a biomarker of oxidative stress. Numerous studies have suggested that elevated urinary MDA levels are significantly associated with the onset and progression of DKD, reflecting ongoing oxidative injury in renal tissues [42, 43]. Heat shock protein 70 (HSP70), a key regulator of the cellular stress response, is also markedly upregulated under hyperglycemic, oxidative, and inflammatory conditions. Increased urinary HSP70‐to‐creatinine ratios have been reported in DKD patients and suggest a decline in renal function. These changes may represent the compensatory protective response of tubular cells to metabolic and oxidative stress [44].

HMGB1, a nuclear nonhistone protein, is passively released during cell stress, necrosis, and apoptosis, which acts as a damage‐associated molecular pattern (DAMP) to promote inflammation. Elevated urinary HMGB1 levels in DKD patients are associated with tubular injury and interstitial fibrosis, potentially through the activation of the TLR4/NF‐*κ*B signaling pathway [45–47]. HMGB1 may serve as a potential diagnostic and prognostic biomarker for DKD. As an important inflammatory alarmin, HMGB1 primarily activates renal inflammatory responses via the TLR/NF‐*κ*B signaling pathway, leading to the upregulation of proinflammatory mediators such as IL‐6 and MCP‐1. Persistent HMGB1‐driven signaling is closely associated with macrophage infiltration, renal tubular epithelial damage, and progressive fibrotic remodeling in DKD [48]. Overall, urinary biomarkers associated with oxidative stress and DNA damage capture multiple aspects of the pathophysiology of DKD, including oxidative DNA damage, lipid peroxidation, reduced antioxidant capacity, mitochondrial dysfunction, and tubular epithelial stress. These markers are of significant value in detecting early redox imbalances in DKD and the transition from metabolic stress to inflammatory activation and fibrotic remodeling.

### 3.2. Inflammation and Immune Response

Interestingly, urinary immunoglobulins, a group of antibodies involved in immune surveillance and inflammatory regulation, are frequently elevated in DKD, reflecting enhanced immune activity and chronic renal inflammation. Among the urinary immunoglobulins, urinary immunoglobulin G (IgG) has been shown to be an independent risk factor for the onset and progression of DKD, highlighting its diagnostic and prognostic potential [49, 50]. However, IL‐6, a multifunctional proinflammatory cytokine, plays a pivotal role in mediating renal inflammation and immune dysregulation in DKD as well. Urinary IL‐6 levels appear to increase progressively with disease severity, particularly in patients with macroalbuminuria, suggesting that urinary IL‐6 may serve as a reliable, noninvasive biomarker of disease activity and progression [51]. IL‐18, another key proinflammatory cytokine, is also actively involved in the renal inflammatory cascade. Research has shown that elevated urinary IL‐18 levels are associated with the development of DKD and atherosclerotic complications in patients with Type 2 diabetes, suggesting its role in both renal and cardiovascular pathophysiology [52].

Moreover, urinary prostaglandin E2 (PGE2), a lipid‐derived inflammatory mediator, participates in regulating renal blood flow, sodium balance, and local immune responses. Increased urinary excretion of PGE2 and its metabolites produced in patients with DKD may reflect underlying vascular inflammation and hemodynamic disturbances [53]. Additionally, the key chemokine, MCP‐1, plays a central role in monocyte recruitment and renal inflammation. The ACCORD trial suggests a strong correlation between the urinary MCP‐1‐to‐creatinine ratio and progressive renal function decline in patients with Type 2 diabetes, which underscores its potential utility in monitoring disease progression and therapeutic response [54, 55]. MCP‐1 is a key inflammatory chemokine in DKD, and it integrates oxidative stress–induced NF‐*κ*B signaling with macrophage recruitment and the subsequent process of interstitial fibrosis and remodeling in the renal tubules [56]. sCD40L, a member of the tumor necrosis factor (TNF) superfamily, is present in immune‐mediated renal injury and fibrotic remodeling in DKD. Elevated urinary sCD40L levels are associated with tubular inflammation, endothelial activation, and glomerular injury, suggesting its role in the disease progression inflammatory–fibrotic axis [57–59]. Further, sCD163, a macrophage‐specific scavenger receptor, is released during monocyte/macrophage activation and is closely associated with proinflammatory cytokine release. Increased urinary sCD163 levels are associated with tubular damage, renal fibrosis, and heightened immune activity in DKD as well, suggesting its possible utility as a marker of macrophage‐driven inflammation and tissue injury [60]. Persistent inflammatory activation in DKD is driven by the activation of the HMGB1/TLR/NF‐*κ*B signaling pathway, which is mediated by oxidative stress. In a hyperglycemic environment, damaged renal tubular epithelial cells release HMGB1, which in turn activates TLR‐dependent signaling pathways and promotes the nuclear translocation of NF‐*κ*B, leading to the transcriptional upregulation of proinflammatory factors such as MCP‐1, IL‐6, IL‐1*β*, and TNF‐*α*. This inflammatory cascade promotes macrophage infiltration and exacerbates immunological damage to the renal tubules. Concurrently, persistent inflammatory signaling interacts with TGF‐*β*1‐dependent pathways, enhancing fibroblast activation and extracellular matrix (ECM) deposition, which collectively lead to the progressive development of tubulointerstitial fibrosis in DKD (Figure 1). Overall, biomarkers of inflammation and immune responses reflect the coordinated activation of cytokine networks, chemokine‐mediated immune cell recruitment, endothelial dysfunction, and fibrosis‐related remodeling processes in DKD. Table 2 summarizes representative urinary biomarkers associated with oxidative stress, inflammation, and fibrosis.

**Table 2:** Oxidative stress‐, inflammation‐, and fibrosis‐associated biomarkers in DKD.

Biomarker name	Function	Clinical significance (EN)	Reference
Oxidative stress biomarkers			
8‐OHdG	Product of oxidative DNA damage	Reflects oxidative DNA injury and renal oxidative stress in DKD	[37]
TBARS	Indicator of lipid peroxidation mediated by free radicals	Reflects the severity of oxidative lipid damage in DKD	[38]
RCDs	Oxidized protein carbonyl derivatives	Indicates protein oxidation and oxidative stress injury in DKD	[38]
TSHGs	Sulfur‐containing antioxidant products involved in redox balance	Reflects antioxidant depletion and oxidative stress status in DKD	[38]
GST	Detoxifying enzyme involved in redox homeostasis	Reflects tubular oxidative stress and DKD‐related renal injury	[39]
WT‐1	Podocyte‐associated transcription factor	Associated with tubular injury and albuminuria progression in DKD	[39]
Mu‐GST	Distal tubular injury‐associated enzyme	Indicates distal tubular damage and oxidative stress injury in DKD	[39]
SOD/SOD3	Antioxidant enzyme scavenging superoxide radicals	Reflects oxidative stress status and disease progression in DKD	[40]
Uric acid	Final product of purine metabolism	Altered urinary excretion reflects oxidative stress, inflammation, and renal dysfunction in DKD	[41]
MDA	End product of lipid peroxidation	Indicates ongoing oxidative injury and progression of DKD	[42]
HSP70	Cellular stress‐response protein	Reflects tubular cellular stress and compensatory protective responses in DKD	[44]
HMGB1	Damage‐associated molecular pattern (DAMP) protein involved in inflammatory signaling	Associated with tubular injury, inflammation, and interstitial fibrosis through TLR4/NF‐*κ*B signaling	[45]
Inflammatory biomarkers			
IgG	Immunoglobulin involved in immune regulation	Reflects chronic renal inflammation and progression of DKD	[50]
IL‐6	Proinflammatory cytokine involved in immune dysregulation	Correlates with disease severity and progression in DKD	[51]
IL‐18	Proinflammatory cytokine participating in inflammatory cascades	Associated with DKD progression and cardiovascular complications	[52]
PGE2	Lipid‐derived inflammatory mediator	Reflects vascular inflammation and hemodynamic disturbances in DKD	[53]
MCP‐1	Chemokine regulating monocyte recruitment and inflammation	Associated with renal inflammation and progressive renal function decline in DKD	[54]
sCD40L	TNF superfamily‐associated inflammatory mediator	Reflects tubular inflammation, endothelial activation, and fibrotic remodeling in DKD	[57]
sCD163	Macrophage activation‐associated scavenger receptor	Indicates macrophage‐driven inflammation, tubular damage, and renal fibrosis in DKD	[60]
Collagen and matrix components			
Fibronectin	Extracellular matrix (ECM) glycoprotein involved in cell adhesion and tissue remodeling	Elevated urinary fibronectin reflects early glomerular structural injury and progressive renal dysfunction in DKD	[61]
Type IV collagen	Major structural component of the glomerular basement membrane and ECM	Reflects glomerulosclerosis, interstitial fibrosis, and progression of DKD	[62]
Laminin	Basement membrane glycoprotein maintaining glomerular and tubular integrity	Increased urinary laminin fragments indicate basement membrane degradation and renal structural damage	[63]
HYAL1	Enzyme involved in extracellular matrix turnover and remodeling	Reflects altered glomerular permeability and interstitial remodeling in DKD	[64]
GAGs	Structural ECM components regulating basement membrane properties	Elevated urinary GAGs indicate glomerular damage and early fibrotic remodeling in DKD	[65]
CS	ECM‐associated glycosaminoglycan involved in matrix metabolism	Reflects ECM accumulation and progression of DKD severity	[66]
Fibrosis‐associated proteins			
MMP‐2	Zinc‐dependent endopeptidase involved in ECM degradation	Reflects renal fibrosis and extracellular matrix remodeling in DKD	[67]
MMP‐9	Matrix‐degrading metalloproteinase involved in tissue remodeling	Associated with renal injury progression and fibrogenic activity in DKD	[68]
MMP‐7	Fibrosis‐associated matrix metalloproteinase	Sensitive biomarker for fibrotic progression and tissue remodeling in DKD	[69]
Renin–angiotensin system (RAS)–related proteins	Regulators of blood pressure, sodium balance, and inflammatory signaling	Reflect intrarenal RAS activation, fibrosis progression, and hypertension in DKD	[70]
uPA	Serine protease involved in plasmin generation and ECM degradation	Indicates interstitial matrix remodeling and fibrotic burden in DKD	[71]
Hydroxyproline	Metabolic product of collagen degradation	Reflects collagen turnover and early fibrotic transformation in DKD	[72]
TGF‐*β*1	Master regulator of fibrosis and EMT signaling	Associated with ECM accumulation, renal fibrosis, declining GFR, and DKD progression	[73]

## 4. Markers of Fibrosis and Tissue Remodeling

A central mechanism underlying fibrosis and structural remodeling in the progression of DKD is the abnormal deposition and impaired degradation of ECM components. Alterations in urinary levels of these ECM components—such as fibronectin, Type IV collagen, laminin, and glycosaminoglycans (GAGs)—and ECM metabolism‐related enzymes, such as hyaluronidase 1 (HYAL1) and urokinase‐type plasminogen activator (uPA), may reflect early structural abnormalities and glomeruli and mesangium fibrotic responses. Furthermore, because urinary biomarkers including matrix metalloproteinases (MMPs), TGF‐*β*1, renin–angiotensin system (RAS)–related proteins, and hydroxyproline are closely associated with tubulointerstitial fibrosis, they likely have a potential role in early disease warning and fibrosis staging. Dynamic monitoring of these fibrosis‐associated urinary biomarkers provides a noninvasive approach for identifying early fibrotic changes and evaluating therapeutic intervention efficacy. Together, all of these biomarkers provide new opportunities for precision management of DKD.

### 4.1. Collagen and Matrix Components

Importantly, fibronectin, a high‐molecular‐weight plasma glycoprotein (~440 kDa), is synthesized primarily by endothelial cells and fibroblasts. It is a key component of the ECM and plays crucial roles in cell adhesion, vascular endothelial integrity, and tissue remodeling. In DKD, fibronectin abnormally accumulates in the glomerular mesangium, which is closely associated with the progressive renal function decline. Further, clinical studies suggest that significantly elevated urinary fibronectin levels in patients with microalbuminuria are a potential early marker of structural injury in DKD [61, 74]. Type IV collagen, a major ECM structural component, is closely associated with renal fibrogenesis. Under diabetic conditions, persistent activation of profibrotic pathways, such as TGF‐*β* signaling, promotes excessive deposition of Collagen IV. This leads to glomerulosclerosis and interstitial fibrosis. Studies suggest that elevated Urinary Type IV collagen levels in diabetic patients, even before the onset of albuminuria, can be used to detect early glomerular structural changes. Further, Urinary Collagen IV levels are positively correlated with the urinary ACR and negatively associated with eGFR; therefore, it may be a promising noninvasive biomarker for fibrosis and assessment of disease progression risk in DKD [62, 75, 76].

Laminin, another key basement membrane glycoprotein, plays an essential role in maintaining glomerular and tubular structural integrity. In DKD, oxidative stress and inflammation induced by chronic hyperglycemia activate proteolytic enzymes that degrade this laminin. The presence of elevated urine laminin fragments suggests the degradation of the basement membrane. This activity could be used as an early biomarker of renal structural damage and disease progression as well [63, 77, 78]. Moreover, HYAL1 also contributes to ECM turnover and remodeling. In a streptozotocin (STZ)‐induced diabetic rat model, urinary HYAL1 activity increased significantly and was positively correlated with albuminuria, suggesting its ability to alter glomerular permeability and its role in interstitial remodeling. Thus, urinary HYAL1 may serve as a novel biomarker for early glomerular injury and fibrotic response in DKD [64]. GAGs, including chondroitin sulfate (CS), are key structural components of the ECM that maintain the glomerular basement membrane physicochemical properties. In diabetic conditions, hyperglycemia disrupts the balance between ECM synthesis and degradation, resulting in the accumulation of GAGs. This can contribute to glomerulosclerosis and interstitial fibrosis. Urinary GAG excretion also increases significantly in the early stages of DKD, often preceding the rise of traditional proteinuria markers. Elevated urinary GAGs are linked not only to glomerular damage but also to DKD progression risk. This highlights GAG′s value as an early and prognostic biomarker [65, 79]. CS is widely distributed in the ECM, and in DKD, chronic hyperglycemia promotes CS accumulation through disturbed ECM metabolism. Further, urinary CS levels are significantly elevated in diabetic patients and are correlated with disease severity, supporting its role in DKD as a marker of ECM remodeling [3, 66].

Overall, urinary biomarkers associated with collagen turnover and ECM components can reflect early abnormalities in matrix homeostasis, basement membrane remodeling, glomerular structural damage, and progressive fibrotic remodeling in DKD. These biomarkers complement traditional assessments of albuminuria by providing additional information on early structural changes and dynamic matrix turnover.

### 4.2. Fibrosis‐Associated Proteins

Urinary MMPs, a family of zinc‐dependent endopeptidases, also play a fundamental role in ECM degradation and remodeling. Among these MMPs, MMP‐2 and MMP‐9 have been extensively studied in the context of DKD. For example, elevated concentrations of these MMPs and enzymatic activities detected in the urine have been seen in affected individuals [67, 68]. Notably, urinary MMP‐7 levels have emerged as particularly sensitive indicators of fibrotic progression and tissue remodeling, offering valuable insights for early detection and longitudinal monitoring in DKD [69]. Increasing evidence suggests that urinary MMP expression profiles correlate strongly with renal injury severity and fibrogenic trajectories, underscoring their potential as prognostic biomarkers.

Importantly, RAS components—including renin, Angiotensin II, and their receptors—play a key mechanistic role in DKD pathogenesis. These molecules regulate blood pressure, sodium reabsorption, and inflammation, all of which contribute to progressive renal injury. Elevated urinary RAS‐related proteins reflect localized activation of the RAS pathway in renal compartments under diabetic conditions. Clinical studies suggest that heightened urinary RAS component levels are significantly associated with DKD disease progression, fibrosis development, and hypertension [3, 70, 80].

Furthermore, in DKD uPA, a serine protease central to ECM catabolism, facilitates the conversion of plasminogen to plasmin. This consequently promotes ECM degradation and peritubular matrix remodeling. Urinary uPA levels are significantly elevated in DKD patients and show dose‐dependent associations with markers of tubular injury and fibrosis progression. These findings suggest that uPA can serve as a valuable indicator of interstitial matrix dysregulation and fibrotic burden in DKD pathophysiology [71]. Hydroxyproline, a metabolic byproduct of collagen degradation, also serves as a direct marker of collagen turnover and fibrotic activity. Increased urinary hydroxyproline levels are seen in diabetic individuals and show a positive correlation = with the severity of microalbuminuria. This suggests that urinary hydroxyproline may provide a noninvasive measure of early fibrotic transformation and matrix remodeling in DKD [72, 81]. TGF‐*β*1 is a master regulator of fibrosis and a key signaling molecule in DKD‐related fibrotic cascades as well. TGF‐*β*1 promotes ECM synthesis, inhibits matrix degradation, and induces epithelial‐to‐mesenchymal transition (EMT). There are strong correlations between elevated urinary TGF‐*β*1 levels and declining GFR, increasing proteinuria, and histopathological fibrosis scores such as interstitial collagen content. Thus, urinary TGF‐*β*1 is widely regarded as a robust prognostic biomarker for renal fibrosis and DKD long‐term disease progression [73]. Chronic inflammation and oxidative stress may act in concert on the TGF‐*β*1 signaling pathway, promoting fibroblast activation, EMT, and ECM deposition in DKD. This process constitutes a key molecular axis driving progressive fibrotic remodeling and tubulointerstitial damage in DKD, as shown in Figure 1.

Fibrosis‐associated urinary proteins can capture multiple aspects of ECM dynamics, including matrix turnover, the activation of profibrotic signaling pathways, and alterations in collagen metabolism during the progression of DKD. These fibrosis‐associated markers, in combination with biomarkers related to oxidative stress and inflammation, aid in the early stratification of fibrosis, prognostic assessment, and monitoring of treatment response. A summary of representative biomarkers is provided in Table 2.

## 5. Metabolic and Endocrine‐Associated Biomarkers

Further, glucose metabolism disorders play central roles in the onset and progression of DKD, and related metabolites and enzymes have emerged as potential urinary biomarkers. Elevated urinary levels of insulin‐like growth factor‐binding protein 3 (IGFBP‐3) degradation products, AGEs, lactate, pyruvate kinase M2 (PKM2), and lactate dehydrogenase (LDH) reflect sustained structural and functional kidney damage caused by glycotoxicity, oxidative stress, and mitochondrial dysfunction. These biomarkers not only provide insights into the metabolic status of the glomeruli and renal tubules but may also predict the risk of DKD progression earlier than conventional clinical indicators. Their combined use is expected to enhance early screening accuracy and the effectiveness of dynamic disease monitoring in clinical practice.

### 5.1. Glucose Metabolism–Related Markers

IGFBPs regulate the bioavailability and activity of insulin‐like growth factors (IGFs). This, in turn, influences key cellular processes such as proliferation, differentiation, and apoptosis. IGFBPs may contribute to renal fibrosis and functional decline in DKD by modulating glomerular and tubular cell behavior. Notably, urinary levels of intact IGFBP‐3 are reduced in DKD patients, whereas its proteolytic fragments are elevated. This pattern suggests that increased IGFBP‐3 degradation may be involved in the pathophysiological mechanisms underlying DKD progression [82]. AGEs are formed through nonenzymatic glycation of proteins and lipids under chronic hyperglycemic conditions as well. These products accumulate in renal tissues and trigger oxidative stress, inflammation, and ECM remodeling. Clinical studies suggest a strong correlation between urinary AGE‐modified protein levels and UACR in diabetic patients. Consequently, urinary AGEs are promising noninvasive biomarkers for monitoring DKD progression, particularly in relation to glomerular injury and oxidative stress [83, 84]. Lactate, a key byproduct of anaerobic glycolysis, accumulates under hypoxic and mitochondrial‐dysfunctional conditions and is commonly seen in diabetic kidneys. Hyperglycemic clamp studies have demonstrated that urinary lactate concentrations increase proportionally with blood glucose levels, indicating their responsiveness to glycemic fluctuations [85]. Mitochondrial dysfunction in renal tubular epithelial cells exacerbates the accumulation of lactate, and elevated urinary lactate is correlated with albuminuria severity, suggesting its role in metabolic dysregulation in DKD development [86].

Furthermore, pyruvate kinase (PK), PKM2, is a critical regulator of aerobic glycolysis. Under hyperglycemic conditions, PKM2 is upregulated in injured renal medullary and tubular epithelial cells. Experimental models of high‐fat diet‐induced diabetes and clinical studies in DKD patients have demonstrated increased urinary PKM2 excretion. These findings support PKM2′s potential utility as an early predictive biomarker of metabolic stress and disease progression in DKD [87]. Additionally, LDH, an enzyme involved in glycolytic metabolism, catalyzes the interconversion of lactate and pyruvate. Its increased urinary activity is associated with declining renal function. Uslu et al. reported that urinary LDH levels rise in parallel with reductions in creatinine clearance and are positively correlated with serum creatinine concentrations. This research reinforces the clinical utility of LDH as a sensitive marker of glomerular and tubular dysfunction in diabetic populations [19, 88]. Collectively, these glucose metabolism–related urinary biomarkers provide a multifaceted view of metabolic stress, mitochondrial dysfunction, and energy imbalance in DKD. Early alterations of these urinary biomarkers offer substantial advantages for noninvasive diagnosis, prognosis, and therapeutic monitoring well before conventional indicators (e.g., albuminuria or eGFR decline) are evident.

### 5.2. Markers Related to Fatty Acid Metabolism and Lipotoxicity

Lipotoxicity driven by dysregulated fatty acid metabolism plays a critical role in the pathogenesis of DKD, particularly in the context of mitochondrial dysfunction, oxidative stress, and inflammation. Urinary biomarkers reflecting fatty acid metabolism offer valuable insights into early renal metabolic stress and injury. For example, acylcarnitines (AcylCNs) are intermediates of fatty acid *β*‐oxidation that provide a direct readout of mitochondrial energy metabolism. Studies suggest that urinary profiles of short‐, medium‐, and long‐chain AcylCNs exhibit stage‐specific alterations in DKD, making them useful for disease staging and longitudinal monitoring [89]. Elevated urinary levels of C14:2‐OH (a hydroxylated long‐chain AcylCN) have been implicated in hyperlipidemia‐induced renal lipotoxicity, highlighting its potential as a predictive marker for lipid‐mediated renal injury as well [90]. In addition, 4‐hydroxy‐2,3‐nonenal (4‐HNE) is a reactive aldehyde generated during lipid peroxidation and serves as a marker of oxidative stress–induced cellular damage. In DKD, increased renal oxidative stress leads to accumulation of 4‐HNE, which can form covalent adducts with proteins and nucleic acids. This consequently impairs cell signaling and function. Elevated urinary 4‐HNE levels have been observed in patients with DKD, suggesting its value as a noninvasive biomarker for oxidative injury and metabolic imbalance [91, 92]. Fatty acid–binding proteins (FABPs), particularly FABP4 and liver‐type fatty acid–binding protein (L‐FABP), are intracellular lipid chaperones involved in fatty acid trafficking, inflammation, and oxidative stress. Elevated urinary FABP4 levels are independently associated with increased UACR and decreased eGFR, indicating its potential to indicate glomerular injury and lipid dysregulation in DKD. Similarly, urinary L‐FABP is a sensitive early biomarker of tubular injury, with elevated levels detectable even before microalbuminuria onset, which supports its role in early diagnosis and monitoring of disease progression [3, 31, 93, 94].

Furthermore, arachidonic acid (AA) and its bioactive derivatives, formed from linoleic acid in membrane phospholipids, are key mediators of inflammation and renal hemodynamics. One such metabolite, tetranor‐prostaglandin E metabolite (tetranor‐PGEM), is significantly elevated in the urine of DKD patients and is associated with both proteinuria severity and renal function decline. Therefore, tetranor‐PGEM may be a novel noninvasive marker for DKD detection and disease tracking [95]. CD36, a transmembrane glycoprotein receptor involved in fatty acid uptake and oxidized LDL binding, also plays an important role in lipid accumulation, inflammation, and renal injury in DKD. Alterations in urinary soluble CD36 reflect disturbances in renal lipid metabolism and are associated with tubular damage and inflammation, suggesting its utility as a lipid‐driven injury biomarker in DKD [96, 97]. Lysophospholipids, including lysophosphatidic acid (LPA) and lysophosphatidylcholine (LPC), are potent lipid signaling molecules that mediate inflammatory responses and fibrosis as well. In DKD, urinary LPA and LPC concentrations are elevated and may reflect underlying lipid metabolic dysregulation and inflammatory injury. Their potential role as urinary biomarkers could enhance early detection and a more thorough mechanistic understanding of lipid‐induced kidney damage in diabetes [98]. Urinary biomarkers associated with glucose and lipid metabolism reflect key aspects of metabolic reprogramming in DKD, including mitochondrial dysfunction, cellular stress induced by lipid overload, oxidative damage, and inflammation‐driven metabolic dysregulation. These changes collectively contribute to the progression of diabetic kidney damage. Representative metabolic, exosomal, and genetic biomarkers discussed in Sections 4 and 5 are summarized in Table 3, which lists their biological functions, pathological relevance, and potential translational significance in DKD.

**Table 3:** Metabolic, exosomal, and genetic biomarkers in DKD.

Biomarker name	Function	Clinical significance (EN)	Reference
Glucose and energy metabolism biomarkers			
IGFBP‐3	Regulator of insulin‐like growth factor (IGF) bioavailability and cellular signaling	Reflects renal fibrosis, tubular dysfunction, and DKD progression	[82]
AGEs	Glycation end‐products formed under chronic hyperglycemia	Associated with oxidative stress, inflammation, glomerular injury, and DKD progression	[84]
Lactate	End product of anaerobic glycolysis and mitochondrial dysfunction	Reflects metabolic stress, hypoxia, and severity of albuminuria in DKD	[85]
PKM2	Glycolytic enzyme regulating aerobic glycolysis	Indicates tubular metabolic stress and progression of DKD	[87]
LDH	Enzyme catalyzing lactate–pyruvate interconversion	Reflects glomerular and tubular dysfunction and declining renal function in DKD	[88]
Lipid metabolism and lipotoxicity biomarkers			
AcylCNs	Intermediates of fatty acid *β*‐oxidation and mitochondrial metabolism	Exhibit stage‐specific alterations and reflect renal lipotoxicity in DKD	[89]
C14:2‐OH	Hydroxylated long‐chain acylcarnitine involved in lipid metabolism	Associated with hyperlipidemia‐induced renal lipotoxicity in DKD	[90]
4‐HNE	Reactive aldehyde generated during lipid peroxidation	Reflects oxidative stress–induced cellular injury and metabolic imbalance in DKD	[92]
FABP4	Intracellular lipid chaperone involved in fatty acid transport and inflammation	Associated with glomerular injury, lipid dysregulation, and DKD progression	[94]
L‐FABP	Lipid‐binding protein involved in tubular fatty acid transport	Sensitive early biomarker of tubular injury and DKD progression	[93]
AA	Polyunsaturated fatty acid involved in inflammatory signaling and renal hemodynamics	Reflects inflammation‐related metabolic dysregulation in DKD	[95]
Tetranor‐PGEM	Urinary metabolite of prostaglandin E2	Associated with proteinuria severity and renal function decline in DKD	[95]
CD36	Transmembrane receptor involved in fatty acid uptake and oxidized LDL binding	Reflects lipid accumulation, tubular inflammation, and renal injury in DKD	[96]
LPA	Bioactive lysophospholipid involved in inflammatory and fibrotic signaling	Indicates lipid metabolic dysregulation and inflammatory injury in DKD	[98]
LPC	Lysophospholipid mediator involved in inflammation and fibrosis	Reflects lipid‐induced renal injury and metabolic imbalance in DKD	[98]
Exosomal and genetic biomarkers			
Urinary exosomes	Extracellular vesicles carrying proteins, lipids, and nucleic acids derived from renal cells	Reflect renal pathophysiological processes and provide noninvasive biomarkers for DKD diagnosis and monitoring	[99]
PCX	Podocyte‐associated glycoprotein involved in maintaining glomerular filtration barrier integrity	Elevated urinary exosomal PCX reflects podocyte injury and glomerular damage in DKD	[100]
miR‐15b	Exosomal microRNA involved in cellular stress regulation	Elevated urinary exosomal miR‐15b is associated with DKD progression	[101]
miR‐34a	MicroRNA involved in apoptosis and inflammatory signaling	Increased urinary exosomal miR‐34a reflects renal cellular injury in DKD	[101]
miR‐636	Exosome‐associated regulatory microRNA	Elevated urinary exosomal miR‐636 may indicate DKD‐associated renal injury	[101]
miR‐21	Fibrosis‐associated microRNA regulating TGF‐*β*/Smad and NF‐*κ*B pathways	Promotes EMT, inflammation, and fibrosis and correlates with albuminuria and glomerulosclerosis in DKD	[102]
miR‐192	MicroRNA regulating TGF‐*β*/Smad signaling and ECM remodeling	Associated with glomerular basement membrane thickening and renal fibrosis in DKD	[103]
miR‐145‐5p	Podocyte injury–related microRNA regulating the RhoA/ROCK pathway	Promotes podocyte apoptosis and reflects glomerular injury in DKD	[104]
miR‐615‐3p	Fibrosis‐associated exosomal microRNA	Correlates with renal injury, inflammation, fibrosis, cystatin C, and TGF‐*β*1 levels in DKD	[105]
miR‐3147	Exosome‐associated microRNA	May participate in DKD progression, although clinical significance remains unclear	[105]
mtDNA	Mitochondrial genetic material released during mitochondrial stress and injury	Reflects mitochondrial dysfunction, oxidative stress, inflammation, and early renal injury in DKD	[106]
TLR9	Innate immune receptor activated by extracellular mtDNA	Mediates mtDNA‐induced inflammatory activation in DKD	[107]
cGAS–STING	Cytosolic DNA‐sensing inflammatory signaling pathway	Contributes to mtDNA‐mediated chronic inflammation and renal injury in DKD	[107]
NLRP3 inflammasome	Inflammasome complex activated by mitochondrial damage signals	Promotes inflammatory responses and progression of DKD	[107]

## 6. Urinary Exosome–Related and mtDNA Biomarkers

With the advancement of liquid biopsy technologies, the ability to detect trace molecular components in urine has become a promising approach for DKD early diagnosis and disease monitoring. In addition to traditional metabolic and protein biomarkers, increasing attention has been directed toward genetic markers such as urinary exosomes and mtDNA. These biomarkers associated with urinary exosomes and mtDNA are also summarized alongside the metabolic biomarkers in Table 3, providing a comprehensive overview of urinary markers related to metabolic, vesicular, and genetic signaling in DKD.

### 6.1. Urinary Exosomes and DKD

Recently, urinary exosomes have gained increasing attention as a novel class of biomarkers in the study of DKD [99]. Exosomes are nanoscale extracellular vesicles, approximately 50–200 nm in diameter, secreted by various cell types. They carry a complex cargo of bioactive molecules, including proteins, lipids, and nucleic acids. Urinary exosomes are derived from multiple renal cell types, including tubular epithelial and interstitial cells, as well as podocytes. This provides a comprehensive snapshot of the renal pathophysiological processes [108, 109]. Growing evidence suggests that urinary exosomes play important roles in the onset and progression of DKD as well [110]. Specific microRNAs (miRNAs) and the proteins they carry can regulate key cellular functions, including inflammation, fibrosis, and apoptosis. For example, elevated levels of urinary exosome podocalyxin (PCX) from DKD patients have been observed, which indicates glomerular injury and may offer diagnostic value in clinical settings [100, 111].

Further, miRNAs within urinary exosomes have emerged as promising noninvasive biomarkers for DKD as well [112]. PCR array analyses have identified significant upregulation of miR‐15b, miR‐34a, and miR‐636 in urinary sediments and exosomes from patients with DKD. These findings have been validated using qRT‐PCR, suggesting their increased expression in exosomes from diabetic individuals [101]. miR‐21 is a well‐studied miRNA associated with fibrosis, which is significantly upregulated in DKD under conditions of chronic hyperglycemia, oxidative stress, and inflammation. A growing body of evidence suggests that miR‐21 plays a key role in renal fibrotic remodeling and persistent inflammatory damage.

Mechanistically, miR‐21 primarily promotes fibrosis by activating the TGF‐*β*/Smad signaling pathway, leading to fibroblast activation, ECM deposition, and the EMT of renal tubular epithelial cells. Concurrently, miR‐21 is also involved in regulating inflammatory signaling pathways, including the activation of NF‐*κ*B, thereby sustaining cytokine production, macrophage infiltration, and chronic tubulointerstitial inflammation [102]. Persistent overexpression of miR‐21 in diabetes may accelerate glomerulosclerosis, tubulointerstitial fibrosis, and progressive structural remodeling of the kidney. During the progression of DKD, miR‐21 exhibits dynamic changes that correlate with the stage of the disease. In the early stages of the disease, a slight elevation of urinary miR‐21 may reflect subclinical inflammatory activation and the initiation of profibrotic signaling pathways prior to overt functional decline. As the disease progresses, the sustained activation of TGF‐*β*1‐dependent pathways further promotes miR‐21 expression, which is associated with worsening proteinuria, a decline in eGFR, increased ECM deposition, and heightened fibrosis. Overall, urinary miR‐21 serves as a noninvasive marker of fibrotic progression and renal function deterioration and may represent a potential therapeutic target for modulating inflammation‐driven fibrotic remodeling in DKD [110].

miR‐192 also regulates TGF‐*β*/Smad signaling and contributes to glomerular basement membrane thickening and interstitial fibrosis. miR‐192 levels in urinary exosomes are increased in DKD patients, especially during the proteinuric stage, which may reflect renal accumulation and excretion dynamics [103, 110]. miR‐145‐5p has been shown to accumulate in urinary exosomes of DKD patients and can also be internalized by podocytes. Once there, it targets *Srgap2* and activates the RhoA/ROCK signaling pathway, inducing podocyte apoptosis. This highlights miR‐145‐5p as a mechanistic and diagnostic marker of glomerular injury in DKD [104]. Further, in a study by Wang et al., expression of miR‐615‐3p and miR‐3147 was analyzed in urinary exosomes. miR‐615‐3p was significantly elevated in DKD patients and strongly correlated with markers of renal injury, inflammation, and fibrosis. In contrast, although miR‐3147 trended upward, no significant clinical correlation was observed between miR‐3147 and DKD. Notably, urinary miR‐615‐3p levels were positively associated with serum cystatin C and plasma TGF‐*β*1, further supporting the utility of miR‐615‐3p as a biomarker for fibrotic progression in DKD [105].

Taken together, urinary exosomes offer several advantages as biomarker sources: They are collected in a noninvasive manner, easily isolated, and abundant in quantity. Their rich content of proteins and miRNAs provides valuable insights into renal injury mechanisms. Thus, urinary exosomes hold great promise for early diagnosis, disease monitoring, and a better understanding of prognosis in patients with DKD [113].

### 6.2. Urine mtDNA

Mitochondrial dysfunction plays a central role in the pathogenesis of DKD, and mtDNA damage is one of the earliest molecular events in this process. mtDNA is highly susceptible to oxidative stress, and under hyperglycemic conditions, mtDNA is prone to copy number reduction, mutation accumulation, and strand breakage. These alterations impair mitochondrial respiratory chain function, disrupt cellular energy metabolism, and induce renal cell apoptosis [106]. Further, damaged mtDNA can be actively released into the extracellular space and excreted in the urine. Extracellular mtDNA functions as a DAMP and can activate multiple innate immune pathways, including Toll‐like receptor 9 (TLR9), cGAS–STING, and the NLRP3 inflammasome. These activities thereby promote a chronic inflammatory state [107]. Unlike blood‐based assays, urinary mtDNA reflects mitochondrial stress directly from renal structures such as tubular epithelial cells and podocytes. For example, studies demonstrate that urinary mtDNA levels are significantly elevated in patients with DKD and are closely associated with decreased eGFR, elevated urinary protein levels, and increased tubular and podocyte injury biomarkers [114].

Notably, one study found that urinary mtDNA levels were increased in diabetic patients, even in the absence of clinical proteinuria. Moreover, the rise in mtDNA also increased levels of urinary proinflammatory cytokines; this suggests that mtDNA elevation may precede conventional proteinuria and serve as an ultra‐early indicator of renal injury and inflammation [115]. Additional studies confirmed that urinary mtDNA levels are significantly higher in DKD patients compared to diabetic patients without nephropathy and are strongly correlated with reduced eGFR and increased albuminuria. These findings support the potential of mtDNA as a noninvasive biomarker for DKD early diagnosis and disease monitoring [114]. Taken together, the evidence here highlights that urinary mtDNA is a sensitive and specific indicator of mitochondrial damage and tubular stress.

Compared to traditional proteinuria markers, urinary mtDNA is noninvasive, stable, and readily accessible. More importantly, it may detect renal injury and mitochondrial dysfunction at an earlier stage, thus providing a powerful tool for early screening and risk stratification in DKD [116]. In the future, combining urinary mtDNA with other novel biomarkers—such as exosomal components and miRNAs—may further enhance diagnostic sensitivity and specificity, enabling improved detection of subclinical DKD. These integrative strategies can be expected to support precise early warning systems and personalized therapeutic interventions for diabetic patients at risk of nephropathy.

## 7. Clinical Application and Translational Challenges of Urinary Biomarkers

Although progress has been made in identifying urinary biomarkers for DKD, significant limitations remain in their clinical translation, particularly with regard to disease‐stage specificity, standardization of analysis, and real‐world application. While these biomarkers offer great promise for early detection and disease management, their clinical value depends largely on their ability to capture subclinical molecular and cellular changes that occur before a marked decline in renal function [117]. The biomarkers discussed in this review collectively reflect the multifactorial nature of DKD, including tubular damage, oxidative stress, inflammatory activation, fibrotic remodeling, metabolic dysregulation, mitochondrial dysfunction, and extracellular vesicle–mediated signaling. These interrelated pathological processes highlight the complexity of the pathogenesis of DKD and suggest that highly coordinated molecular interactions underlie disease progression [22, 118]. Consequently, future biomarker research should not only focus on identifying new personalized biomarkers but also emphasize monitoring strategies for different disease stages, multibiomarker panel analysis, and precision medicine approaches based on multiomics. Against this backdrop, strengthening the standardization of biomarkers, enhancing clinical translation capabilities, and integrating artificial intelligence (AI)–based predictive models are expected to further advance the personalized management of DKD [119, 120].

### 7.1. Clinical Value and Limitations of Current Biomarkers

Although significant progress has been made in research into urinary biomarkers, there remain some important limitations in DKD that restrict their widespread clinical application. Many biomarkers lack sufficient specificity when used in isolation and may be influenced by factors such as systemic inflammation, metabolic status, medication, age, and declining renal function [117]. Furthermore, differences in urine collection methods, sample storage procedures, testing platforms, and threshold settings lead to variability between studies and limit the reproducibility of results. Coupled with the heterogeneity of the clinical presentation of DKD—particularly the increasing prevalence of nonproteinuric DKD—this further complicates the interpretation of biomarkers [118].

Importantly, many urinary biomarkers exhibit changes associated with the stage of DKD as the condition progresses. Biomarkers associated with tubular stress and oxidative damage, such as KIM‐1 and NGAL, may be elevated during the early stages of glomerular hyperfiltration and tubulointerstitial damage, whereas biomarkers associated with fibrosis and mitochondrial dysfunction become more pronounced during the later stages of kidney damage and renal failure [117]. These findings indicate that different biomarkers may have different clinical values at different stages of DKD, highlighting the importance of selecting specific biomarkers for different stages in disease monitoring and prognosis assessment.

Therefore, although urine biomarkers offer promising prospects for early diagnosis and dynamic monitoring, before these biomarkers can be fully applied in clinical routine practice, large‐scale multicenter prospective studies and standardized detection protocols still need to be carried out [117, 118].

### 7.2. Combined Biomarker Strategies and Clinical Translation

Since DKD is a highly heterogeneous disease involving multiple pathological pathways, relying solely on a single biomarker is insufficient to fully reflect the overall progression of the disease. Different biomarkers represent different biological processes, including tubular damage, inflammation, oxidative stress, fibrosis, metabolic disorders, and mitochondrial dysfunction. Therefore, an integrated biomarker combination may be more capable of comprehensively assessing the status and progression of DKD compared to using a single biomarker alone [121, 122].

Recent studies have shown that the combined use of biomarker strategies can significantly improve diagnostic accuracy and the level of disease stratification. For instance, simultaneously detecting markers of renal tubular damage (such as KIM‐1 and NGAL) and inflammatory markers (such as MCP‐1 and IL‐6) can help identify patients with subclinical renal tubulointerstitial damage at an early stage before there is a significant decline in eGFR or the appearance of significant proteinuria [117]. Furthermore, the LC‐MS/MS proteomics study combined with machine learning algorithms demonstrated that the multiprotein classifier could accurately distinguish simple diabetes from DKD and further differentiate different stages of DKD with high diagnostic accuracy [119, 123]. Metabolomics and proteomics analyses also revealed stage‐specific metabolic changes related to oxidative stress, amino acid metabolism, and mitochondrial dysfunction, significantly improving the ability to distinguish early DKD from advanced DKD [121, 122, 124]. Furthermore, through metabolomics research using machine learning, a set of urine metabolite profiles was discovered that can distinguish different stages of DKD and predict the decline in renal function [120].

The extracellular vesicles in urine and the biomarkers related to exosomes also show great potential as noninvasive diagnostic tools due to their ability to better reflect the physiological and pathological states of renal cells. Recent proteomic studies on exosomes have revealed that the biomarkers related to exosomes in urine can distinguish between DKD and non‐DKDs and are closely associated with renal function decline and hyperglycemic state. These findings suggest that combining exosome‐related biomarkers with traditional urine biomarkers is expected to further enhance the specificity of the disease and promote noninvasive differential diagnosis [125, 126].

Despite these encouraging research results, clinical translation still faces several significant challenges. The lack of standardized procedures for urine collection, extracellular vesicle isolation, and proteomics and metabolomics analysis, as well as biomarker quantification, leads to poor reproducibility among different studies [118, 125, 126]. Furthermore, the validation of many candidate biomarkers in large‐scale and racially diverse patient populations is still insufficient. The relatively high cost and complex technical requirements of high‐throughput omics technologies also limit their widespread application in clinical settings. Therefore, future research should focus on improving the standardization level, optimizing the combination of biomarkers at different stages, and establishing clinically feasible and economically efficient detection strategies [127].

### 7.3. Multiomics and AI‐Assisted Precision Medicine

With the rapid development of high‐throughput omics technologies, including genomics, transcriptomics, proteomics, metabolomics, lipidomics, and single‐cell sequencing technologies, the research on DKD biomarkers has entered a new stage of integrated molecular phenotype analysis [127]. Multigeneomic methods can comprehensively reveal the complex molecular mechanisms underlying the progression of DKD and help identify novel biomarker networks related to early kidney damage, disease heterogeneity, and treatment responses. The integrated multigeneomic analysis shows that during the progression of DKD, coordinated changes occur in processes such as inflammatory signal transduction, oxidative stress pathways, mitochondrial dysfunction, ECM remodeling, immune activation, and metabolic reprogramming [118, 122].

Recent studies that integrated proteomics, metabolomics, and peptideomics have shown that multiomics approaches can enhance the early detection and disease stratification capabilities of DKD [118, 122]. The combination of multiomics analysis and machine learning algorithms has significantly enhanced the ability to distinguish early DKD, overt DKD, and high‐risk diabetic patients before their clinical symptoms deteriorate [119, 120, 123]. Similarly, the integrated urine proteomics study identified a biomarker classifier that can distinguish different stages of DKD and is used to monitor patients at high risk of progression [119, 123].

AI and machine learning methods are increasingly being applied to the research of DKD biomarkers. By integrating multiomics data and clinical variables through AI, it helps to optimize the selection of biomarkers, disease stratification, and personalized risk prediction [119, 127]. The machine learning‐based model can also enhance the ability to distinguish different stages of DKD, predict rapid deterioration of renal function, and evaluate treatment responses [120]. Furthermore, extracellular vesicles in urine and biomarkers related to exosomes are gradually emerging as promising noninvasive liquid biopsy tools, as they may provide a more accurate reflection of the physiological and pathological states of renal cells [125].

Overall, future research on DKD biomarkers should not only focus on discovering new biomarkers but also integrate multipanel biomarker sets, multiomics technologies, mechanism pathway analysis, and AI‐assisted predictive modeling into a clinically applicable precision medicine framework [118]. These advancements may significantly improve the early diagnosis, dynamic monitoring, prognosis assessment, and individualized management of patients with DKD.

## 8. Conclusions and Perspectives

**Figure 2:**
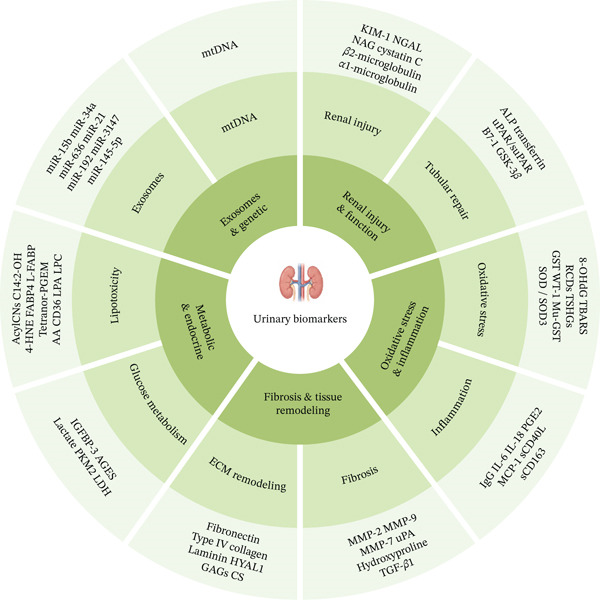
Summary diagram of all urinary biomarkers associated with DKD. This schematic diagram summarizes the major categories of urinary biomarkers discussed in this review. The circular arcs represent five biomarker groups: renal injury and functional biomarkers, oxidative stress and inflammation‐related biomarkers, fibrosis and tissue remodeling biomarkers, metabolic and endocrine‐associated biomarkers, and urinary exosome–related and mitochondrial DNA biomarkers. Different colors indicate corresponding subcategories, including glomerular and tubular injury, tubular metabolism and repair, oxidative stress and DNA damage, inflammation and immune response, collagen and matrix components, fibrosis‐associated proteins, glucose metabolism, fatty acid metabolism and lipotoxicity, urinary exosomes, and urinary mitochondrial DNA. Representative biomarkers are arranged along each arc to provide an overview of their pathological relevance and potential clinical value in DKD diagnosis, disease monitoring, and risk stratification.

Urinary biomarkers offer a noninvasive and dynamic approach to the early detection, risk stratification, and long‐term monitoring of DKD. As shown in Figure 2, these biomarkers encompass multiple pathological dimensions, including renal injury, oxidative stress, inflammatory activation, fibrotic remodeling, metabolic disorders, lipotoxicity, and exosome‐ or genetic‐related alterations. This comprehensive biomarker profile highlights the multifactorial and heterogeneous nature of DKD and supports the view that composite biomarker panels may be superior to single‐marker strategies in disease assessment.

Despite these advantages, clinical translation faces several obstacles, including limited disease specificity, significant variability across different platforms, a lack of standardized reference thresholds, and insufficient prospective validation. Future research should prioritize multicenter cohort validation, the development of biomarker panels tailored to disease staging, the integration of multiomics datasets, and the application of AI‐based predictive modeling techniques. These approaches will help to translate urinary biomarkers into tools for clinical precision diagnosis, personalized monitoring, and early intervention in DKD.

## Author Contributions

Yujie Jin, Yan Ma, Yan Yao, Mengru Wang, and Chunchen Ni contributed equally to this work and share first authorship. Lizhuo Wang and Jialin Gao share corresponding authorship. Conceptualization: Yujie Jin, Lizhuo Wang, and Jialin Gao. Methodology: Yujie Jin, Yan Ma, Yan Yao, Mengru Wang, and Chunchen Ni. Investigation (literature search and study selection): Yujie Jin, Yan Ma, Yan Yao, Mengru Wang, and Chunchen Ni. Data curation: Shujuan Shang, Yongxin Cui, Xinyu Wang, and Ye Ling. Formal analysis: Shujuan Shang, Yongxin Cui, Xinyu Wang, and Ye Ling. Visualization: Yumeng Sun and Qirui Pei. Software (if applicable/or figure preparation): Yumeng Sun and Qirui Pei. Validation: Shiqiang Liu. Writing—original draft: Yujie Jin, Yan Ma, Yan Yao, Mengru Wang, and Chunchen Ni. Writing—review and editing: Shiqiang Liu, Lizhuo Wang, and Jialin Gao. Supervision: Lizhuo Wang and Jialin Gao. Project administration: Lizhuo Wang and Jialin Gao.

## Funding

This study was funded by the Major Program of Anhui Provincial Health and Medical Research Project (2024BAC50001), the National Natural Science Foundation of China (82370808), the Clinical Medical Research Transformation Project of Anhui Province (202527c10020029, 202527c10020025, 202527c10020040, and 202527c10020017), the Funding of “Unveiling the List and Appointing the Best” Project from Yijishan Hospital (2023‐3‐07), the Anhui Provincial Higher Education Institutions Innovation Team Project (2025AHGXZK10020), and the China Medical Foundation (2025CMFC11).

## Disclosure

All authors have read and approved the final version of the manuscript. The corresponding author, Jialin Gao, had full access to all of the data in this study and takes complete responsibility for the integrity of the data and the accuracy of the data analysis.

## Ethics Statement

Ethical approval was not required for this study because this is a scoping review based solely on previously published literature and does not involve human participants or animals.

## Conflicts of Interest

The authors declare no conflicts of interest.

## Data Availability

Data sharing is not applicable to this article as no datasets were generated or analyzed during the current study.
